# Mapping a gene for rheumatoid arthritis on chromosome 18q21

**DOI:** 10.1186/1753-6561-1-s1-s18

**Published:** 2007-12-18

**Authors:** William Tapper, Andrew Collins, Newton E Morton

**Affiliations:** 1 Human Genetics Division, University of Southampton, Southampton General Hospital, Tremona Road, Southampton, Hampshire SO16 6YD. UK

## Abstract

Although single chi-square analysis of the North American Rheumatoid Arthritis Consortium (NARAC) data identifies many single-nucleotide polymorphisms (SNPs) with *p*-values less than 0.05, none remain significant after Bonferroni correction. In contrast, CHROMSCAN evades heavy Bonferroni correction and auto-correlation between SNPs by using composite likelihood to model association across all markers in a region and permutation to assess significance. Analysis by CHROMSCAN identifies a 36-kb interval that includes the most significant SNP (msSNP) observed in a 10-Mb target suggested by linkage. Unexpectedly, stratification by gender and age of onset shows that association evidence comes almost entirely from females with age of onset less than 40. Combining evidence from a meta-analysis of linkage studies and three subsets of the NARAC data provides significant evidence for a determinant of rheumatoid arthritis in a 36-kb interval and illustrates the principle that estimates of location and its information are more powerful than estimates of *p*-values alone.

## Background

Initially, linkage mapping dealt with rare and highly penetrant genes. Without cytogenetic assignment, the preferred strategy was segregation analysis to determine all relevant parameters except recombination, followed by linkage analysis to determine recombination frequency [1]. Complex inheritance with uncertain segregation parameters proved much more difficult, giving rise to many unconfirmed claims based on microsatellites and leading to meta-analysis without point locations [2]. The HapMap project provides dense SNPs that can be used to localize causal loci with or without pedigrees. This procedure, called *association mapping*, revolutionized identification of disease genes. Recent developments of linkage disequilibrium units (LDU), composite likelihood, control of auto-correlation, and meta-analysis are incorporated into the CHROMSCAN program [3,4] to increase its precision for association mapping. Here we use these methods to establish the location and weight of evidence for a gene predisposing to rheumatoid arthritis.

## Methods

### Data preparation

The data, provided by NARAC (North American Rheumatoid Arthritis Consortium) consist of 2300 single-nucleotide polymorphisms (SNPs) in a 10-Mb region of 18q21 with linkage evidence in U.S. and French scans [5]. Illumina genotyped these markers in 460 cases and 460 controls, matched for age and gender, from New York. The genotypic data for controls were screened and 7 SNPs with $\chi_{1}^{2}$ > 10 for the Hardy-Weinberg test [6] were removed, leaving 2293 to be analyzed. CHROMSCAN requires SNPs to be located on both physical and LDU scales. Physical locations were taken from build 35 of the human genome sequence. Unlike physical maps, study-specific and various LDU maps are available, corresponding to the four HapMap samples separately and combined (CEU, CHB, JPT, YRI, and cosmopolitan). The LDU map with the highest SNP density and population attributes closest to the experimental data should be optimal. We therefore used LDU locations relative to the CEU HapMap data with a density of 1 SNP per 863 bp compared to 1 SNP per 4139 bp in the NARAC data. We also used the kilobase map to determine the robustness and power of LDU maps compared with physical maps.

### LDU map construction

The theory for constructing LDU maps has been described [7]. Briefly, the LDU distance for the *i*^th ^SNP interval is given by *ε*_*i*_*d*_*i*_, where *ε*_*i *_describes the exponential decline of association with physical distance *d*_*i *_in kb. Values of *ε*_*i *_are estimated by composite likelihood that fits the Malecot model [8] to multiple pairwise diplotype data. The Malecot equation, given by $\rho =(1-L)Me^{-\sum \varepsilon_{i}d_{i}}+L$, uses additional parameters to describes association at the last major bottleneck (*M*), and residual association at large distance (*L*) to predict rho (*ρ*), the probability of association.

### Association mapping

The CHROMSCAN program [3] uses a model similar to LDU maps except the exponential term is replaced by *ε*Δ(*S*_*i *_- *S*) to estimate the location (*S*) of a disease gene, where *S*_*i *_is the location of the *i*^*th *^marker in kilobases or LDU. The Kronecker Δ is used for map direction and assures a correct sign, with Δ = 1 if *S*_*i *_≥ *S *or -1 if *S*_*i *_<*S*. To calculate the expected association with distance, *z*_*i*_, the model becomes $z_{i}=(1-L)Me^{-\varepsilon \Delta (S_{i}-S)}+L$, where *M *is diminished by complex inheritance and *L *is the association at large distance. The observed association ${\overset{⌢}{z}}_{i}$ is determined by a 2 × 2 table between affection status and the two alleles of each SNP to give $\left|\begin{array}{cc} a & b\\ c & d \end{array}\right|$ and ${\overset{⌢}{z}}_{i}=\frac{\left(ad-bc\right)}{\left(a+b)(b+d\right)}$, where *ad *- *bc *≥ 0 and *b *≤ *c *is ensured by rearrangement of columns and rows [9]. Given the observed associations ${\hat{z}}_{i}$, the Malecot parameters are estimated iteratively using composite likelihood, which evades a heavy Bonferroni correction by combining information over all loci within a region as $\Lambda ={\sum K_{i}({\hat{z}}_{i}-z_{i})}^{2}$, where ${\hat{z}}_{i}$ and *z*_*i *_are the observed and expected association values, respectively, at the *i*^th ^SNP. Their squared difference is weighted by information (*K*_*i*_) which is estimated as: $K_{i}=\chi_{1}^{2}/{\hat{z}}_{i}^{2}$, where $\chi_{1}^{2}$ is the Pearson $\chi_{1}^{2}$ from the 2 × 2 table.

Sub-hypotheses of the Malecot model are used to test for a causal polymorphism. Model A, which estimates none of the parameters and uses *M *= 0 with predicted *L *[10], is taken as the null hypothesis *H*_0 _in which there is no association between affection status and SNPs. Model D estimates *M*, *S*, and *L*. Therefore the Λ_A _- Λ_D _comparison tests for a disease determinant at location *S*. For both models, *ε *is fixed to 1 for the LDU map and to a value of *ε *determined from pairwise marker-by-marker association data for the kilobase map. In order to account for autocorrelation between SNPs as a result of LD, the significance of evidence is determined by a rank-based permutation test [3].

Three separate analyses of the data were performed by CHROMSCAN. The first is a preliminary screen of the entire 10-Mb bin, which is divided into 18 nonoverlapping regions, each with at least 30 SNPs and covering at least 10 LDUs. To determine accurate levels of significance, the number of permutation replicates must approach the actual level of significance so that interpolation of the variance under *H*_1 _is reliable. To minimize computation time, the initial analysis was restricted to 100 replicates. Significant regions identified by the initial screen were re-analyzed separately using 1000 and 5000 replicates in order to verify convergence. To demonstrate the power of LDU maps, this analysis was repeated using the kilobase map and two estimates of the exponential decline *ε *derived from the significant region and the 10-Mb region [11]. The risk for rheumatoid arthritis is elevated in females, especially with late onset (≥35–≤60) [12]. Our third analysis therefore stratified cases into three groups corresponding to males, females with onset ≤39, and females with onset ≥40. The partition of females around an onset age of 40 was chosen to give approximately equal numbers of 'early' and 'late' onset cases. Unaffected controls for the three groups were all males (with similar age and total number of individuals as affected males), and females divided by current age to give similar total numbers of individuals as cases, respectively. This analysis was restricted to significant regions from the initial screen and used 5000 replicates.

## Results

### Association mapping

Single chi-square analyses of the 10-Mb region identifies 125 SNPs with *p *< 0.05, none of which reach significance after Bonferroni correction (0.05/2293). The initial screen by CHROMSCAN divides the 18q21 bin into 18 nonoverlapping regions. Although the most significant SNP (msSNP, rs3745064) occurs in region 6, the next msSNP in region 11 is deceptively close in terms of significance, and several other regions contain suggestive SNPs (Table 1). In contrast, the composite likelihood approach, which models association across all markers in a region, identifies region 6 as the only significant region (*p *= 0.01259). The intensive screen of region 6 identified a large increase in significance between 100 and 1000 replicates, which is attributed to the relationship between number of replicates and significance, while the small decrease in significance between 1000 and 5000 replicates suggests that convergence has been achieved (Table 2). These analyses estimate a causal locus (*S*) at 53308 kb.

**Table 1:** Regions screened with 100 replicates

Region	No. SNPs	First kb	Last kb	$\chi_{1}^{2}$ for msSNP	Composite likelihood		
					
					$\chi_{1}^{2}$	LOD_1_	*p*-Values
1	256	48896	49928	6.58	0.05948	0.01291	0.80701
2	238	49935	51009	11.18	0.00691	0.00150	0.93358
3	197	51010	51919	10.83	2.22538	0.48323	0.13584
4	132	51926	52398	4.42	0.05000	0.01086	0.82274
5	241	52398	53262	5.90	0.00011	0.00002	0.99158
6	113	53262	53596	12.25	6.22830	1.35246	0.01259
7	74	53599	53919	6.84	2.95054	0.64070	0.08592
8	38	53920	54006	1.75	0.78229	0.16987	0.37637
9	103	54008	54448	6.64	1.71241	0.37185	0.19074
10	49	54496	54974	5.23	0.50780	0.11027	0.47592
11	70	54977	55285	12.06	0.34910	0.07581	0.55438
12	60	55286	55381	10.89	0.00011	0.00002	0.99147
13	89	55382	55634	4.24	0.13215	0.02870	0.71588
14	29	55641	55838	2.42	0.05010	0.01088	0.82258
15	134	55839	56399	5.66	0.97584	0.21190	0.32321
16	209	56408	57248	3.87	0.00166	0.00036	0.96744
17	55	57253	57545	9.51	1.51557	0.32910	0.21834
18	200	57548	58415	3.86	1.58390	0.34394	0.20826

**Table 2:** Intensive screening of region 6

No. replicates	Map^a^	*ε*	*S*		*K*	SE	$\chi_{1}^{2}$	LOD_1_	*p*
								
			kb	LDU					
100	LDU	1.000	53308.370	1195.126	41.233	0.1557	6.2283	1.3525	0.0126
1000	LDU	1.000	53308.370	1195.126	44.329	0.1502	9.6876	2.1036	0.0019
5000	LDU	1.000	53308.370	1195.126	36.611	0.1589	8.3245	1.8076	0.0039
1000	kb	0.021	53306.868	1195.126	0.0195	7.1529	7.2178	1.5673	0.0072
1000	kb	0.031	53307.748	1195.126	0.0367	5.2220	7.2910	1.5832	0.0069

^a^Values of information (*K*) and standard error (SE) are relative to *S*_LDU _and *S*_kb_, indicated by the map column, when using the LDU and kb map, respectively.

The CHROMSCAN analysis of region 6 was repeated using the kilobase map so that its performance can be compared with the LDU map. Using a kilobase map requires specification of the exponential decline *ε *[11]. Two values of *ε*, corresponding to the 10 Mb interval (0.021) or region 6 alone (0.031), were investigated. Despite the large difference between *ε *values for the kilobase map, the significance level and location were almost identical. However, the ratios of $\chi_{1}^{2}$ indicate that the kilobase maps have a relative efficiency of 75% compared with an LDU map at 1000 replicates (Table 2).

Because King et al. [12] demonstrated that the risk for rheumatoid arthritis is elevated in females, especially with late onset, we stratified cases into three groups according to sex and age of onset. The effect of this stratification is highly suggestive despite its crudeness (Table 3) and small sample sizes. Females with onset ≤39 account for most of the association. The other two classes give such small chi-square values that they would undoubtedly be assigned to other regions if the partition test had not been restricted to region 6 on the pooled evidence. However, when considering region 6 alone, there is remarkable agreement between point estimates for 'early' and 'late' onset females and those from males. At this time it is impossible to say whether this consistency is caused by imperfectly divided onset groups or a small effect at late age.

**Table 3:** Stratification by gender and age of onset (5000 replicates)

Sex	Age of onset	Case/Control		*S*		*K*	SE	$\chi_{1}^{2}$	LOD_1_	*p*
									
				kb	LDU					
M	-	95	95	53308.370	1195.126	3.13	0.57	0.0299	0.007	0.8624
F	≤39	193	192	53308.370	1195.126	47.3	0.15	11.844	2.575	0.0006
F	≥40	170	173	53262.482	1194.474	0.04	5.18	0.0183	0.004	0.8589

### Linkage

Choi et al. [13] reported a meta-analysis of four linkage studies with microsatellites in a 10-Mb bin of chromosome 18. The results from this study were reported as *p*-values without estimates of location or standard errors. Without this information, the power for meta-analysis is reduced because the sum of two $\chi_{1}^{2}$ values must be converted back to $\chi_{1}^{2}$ and LOD_1 _instead of weighting estimates of location by their information. Perhaps because of this inefficiency, the combined LOD_1 _from this meta-analysis is 1.542, well below the conventional value of 3 for asserting significance. The corresponding *p*-value in large-sample theory is 0.007714, providing strong but inconclusive evidence for localization in the 18q21 region. Despite its limitations, linkage contributes evidence that should not be ignored.

### Joint significance of linkage and association

The simplest meta-analysis is based on *n *independent samples, the *i*^th ^of which contributes a *P*_*i *_value that on the null hypothesis is uniformly distributed. Then -2 ln *P*_*i *_would be distributed as $\chi_{2}^{2}$, with $\chi_{2n}^{2}=-2\sum \ln P_{i}$. This is the only test applicable to data that do not provide an estimate of location *S*_*i *_and information *K*_*i*_, but has three disadvantages; first, equal weight is given to samples with different standard errors; second, there is no test of homogeneity; and third, there is no point estimate to become more precise as *n *increases. As a consequence, much information is lost. Accepting these limitations and assuming accuracy of the *P *estimates, Table 4 shows that combining pooled association with linkage provides suggestive evidence to assign a gene for rheumatoid arthritis to the 18q21.31 interval. The LOD_1 _with no Bonferroni correction is 2.676 for linkage and pooled association. When location and information weight are available, the evidence for association is combined by determination of the difference between $\Sigma \chi_{1}^{2}$ with n degrees of freedom and $\Sigma K_{i}{\left({\hat{S}}_{i}-\bar{S}\right)}^{2}$, which tests for heterogeneity with *n *- 1 degrees freedom where $\bar{S}=\Sigma K_{i}S_{i}/\Sigma K_{i}$. When the stratified association samples are combined in this manner, the heterogeneity test is negligible. As expected, power is increased when pooled with linkage (LOD_1 _= 3.401, *p *= 0.000076). Even with conservative adjustment of the *p*-value to account for the 18 regions tested by association (18*0.000569), and despite strong although not formally significant, evidence from linkage for at least one causal gene in the 18 regions, the meta-analysis is supportive (LOD_1 _= 2.327, *p *= 0.001062). We conclude that evidence for region 6 is probative, with linkage and association both providing critical evidence despite lack of a point estimate and information weight for linkage.

**Table 4:** Meta-analysis of association (5000 replicates) and linkage

Study	Source	$\chi_{1}^{2}$	LOD_1_	*p*
1	Choi et al. [13]	7.101	1.542	0.007714
2	Association (region 6)	8.3245	1.808	0.003916
Pooled 1 and 2		12.326	2.676	0.000447
				
Association (stratified) using *P*		7.0896	1.539	0.007762
Association (stratified) using *S*, *K*		11.875	2.579	0.000569
Pooled with 1		15.662	3.401	0.000076
				
Association (stratified) using *S*, *K *and adjusted		6.5944	1.432	0.010242
Pooled with 1		10.718	2.327	0.001062

## Discussion

This application demonstrates that CHROMSCAN is a powerful approach for gene mapping in complex inheritance, which is applicable to meta-analysis. Obvious extensions include identification of a causal locus and more precise definition of the phenotype associated with it. The 95% confidence interval, given by *S *± 1.96 (SE), covers 36 kb between 53296 and 53332 kb and includes the msSNP rs3745064. Although no described genes are within this region, it does include four human mRNAs from GenBank: CR590917, AK021217, AK124558, and BC01314, all to the left of point estimate (S). Of these, CR590917 appears to be the most interesting because it is expressed within T cells and could therefore conceivably affect risk for rheumatoid arthritis. Finally, geneid [14] and Genscan [15] predict a similar gene, which is the closest annotated sequence to the point estimate (*S*). However, nothing is known about the function of this gene and its reliability is questionable. The fascinating directions revealed by these findings have yet to be explored. Ultimately, interaction with other contributing loci and environmental factors will be recognized and, more importantly, locus-specific treatment will be found.

Recent papers testify to growing interest in meta-analysis, looking backward to linkage rather than forward to association mapping. Rank permutation provides a valid significance test, but the genome search meta-analysis (GSMA) that uses regional assignment with arbitrary weights cannot give a reliable estimate of effect and therefore has low power for estimating point location and detecting heterogeneity [16,17]. Most of the few papers on association mapping assume family data rarely feasible for diseases of late onset and are restricted to single markers without composite likelihood to estimate both location *S *and its information *K*. One manuscript presented in GAW15 that used meta-analysis without those estimates failed to detect the strong signal on chromosome 18q demonstrated by composite likelihood [18].

## Competing interests

The author(s) declare that they have no competing interests.
